# Bayesian inference of transmission chains using timing of symptoms, pathogen genomes and contact data

**DOI:** 10.1371/journal.pcbi.1006930

**Published:** 2019-03-29

**Authors:** Finlay Campbell, Anne Cori, Neil Ferguson, Thibaut Jombart

**Affiliations:** 1 MRC Centre for Global Infectious Disease Analysis, Department of Infectious Disease Epidemiology, School of Public Health, Imperial College London, United Kingdom; 2 Department of Infectious Disease Epidemiology, London School of Hygiene & Tropical Medicine, London, United Kingdom; 3 UK Public Health Rapid Support Team, London, United Kingdom; Yale School of Public Health, UNITED STATES

## Abstract

There exists significant interest in developing statistical and computational tools for inferring ‘who infected whom’ in an infectious disease outbreak from densely sampled case data, with most recent studies focusing on the analysis of whole genome sequence data. However, genomic data can be poorly informative of transmission events if mutations accumulate too slowly to resolve individual transmission pairs or if there exist multiple pathogens lineages within-host, and there has been little focus on incorporating other types of outbreak data. We present here a methodology that uses contact data for the inference of transmission trees in a statistically rigorous manner, alongside genomic data and temporal data. Contact data is frequently collected in outbreaks of pathogens spread by close contact, including Ebola virus (EBOV), severe acute respiratory syndrome coronavirus (SARS-CoV) and *Mycobacterium tuberculosis* (TB), and routinely used to reconstruct transmission chains. As an improvement over previous, ad-hoc approaches, we developed a probabilistic model that relates a set of contact data to an underlying transmission tree and integrated this in the *outbreaker2* inference framework. By analyzing simulated outbreaks under various contact tracing scenarios, we demonstrate that contact data significantly improves our ability to reconstruct transmission trees, even under realistic limitations on the coverage of the contact tracing effort and the amount of non-infectious mixing between cases. Indeed, contact data is equally or more informative than fully sampled whole genome sequence data in certain scenarios. We then use our method to analyze the early stages of the 2003 SARS outbreak in Singapore and describe the range of transmission scenarios consistent with contact data and genetic sequence in a probabilistic manner for the first time. This simple yet flexible model can easily be incorporated into existing tools for outbreak reconstruction and should permit a better integration of genomic and epidemiological data for inferring transmission chains.

## Author summary

Reconstructing the history of transmission events in an infectious disease outbreak provides valuable information for informing infection control policy. Recent years have seen considerable progress in the development of statistical tools for the inference of such transmission trees from outbreak data, with a major focus on whole genome sequence data (WGS). However, complex evolutionary behavior, missing sequences and the limited diversity accumulating along transmission chains limit the power of existing approaches in reconstructing outbreaks. We have developed a methodology that uses information on the contact structures between cases to infer likely transmission links, alongside genomic and temporal data. Such contact data is frequently collected in outbreak settings, for example during Ebola, HIV or Tuberculosis outbreaks, and can be highly informative of the infectious relationships between cases. Using simulations, we show that our contact model effectively incorporates this information and improves the accuracy of outbreak reconstruction even when only a portion of contacts are reported. We then apply our method to the 2003 SARS outbreak in Singapore and describe the range of transmission scenarios consistent with genetic data and contact data for the first time. Our work suggests that, whenever available, contact data should be explicitly incorporated in outbreak reconstruction tools.

## Introduction

Inferring chains of transmission in an infectious disease outbreak can provide valuable epidemiological insights into transmission dynamics, which can be used to guide infection control policy. For example, reconstructed outbreaks have been used to identify drivers of ongoing infection [1], characterize heterogeneous infectiousness in a population [2], evaluate the effectiveness of interventions [3] and determine transmission mechanisms [4]. Consequently there has been increased interest in developing statistical and computational tools for inferring such ‘transmission trees’ from various types of data, including times of symptom onset, contact tracing data, spatial data and, increasingly frequently, pathogen whole genome sequence (WGS) data [5–13].

Most state of the art outbreak reconstruction tools aim at approximating a posterior distribution of likely transmission trees in a Bayesian MCMC framework. Two major approaches have emerged, which can be defined by their treatment of genetic data [14]. The ‘*pairwise approach*’ begins with a model of disease transmission and attaches to this a genetic model that describes the pairwise genetic distance between putative transmission pairs [6–9]. The ‘*phylogenetic approach*’ uses genetic data to infer the unobserved history of coalescent events between sampled pathogen genomes in the form of a phylogenetic tree and infers transmission trees consistent with this phylogeny using epidemiological data. Such methods either use a fixed phylogeny inferred *a priori* [10,15] or jointly infer the phylogeny alongside the transmission tree itself [11–13].

These methodologies differ in their ability to identify unobserved or imported cases, accurately describe evolutionary behavior in the presence of multiple dominant strains within-host or incomplete transmission bottlenecks and accommodate multiple genetic sequences per host. However, a notable similarity between these studies is the fact that they generally only consider temporal and genetic data. Accordingly, such approaches rely heavily on highly informative genetic sequence data for identifying likely transmission pairs, as temporal data is generally consistent with a large number of potential ancestries [16].

However, WGS are not always informative of the transmission route of an epidemic. Firstly, genetic diversity across most outbreaks is low and a significant portion of genetic sequences expected to be identical [17], most prominently if the pathogen genome is small (e.g. human influenza [18]), the mutation rate low (e.g. *Mycobacterium tuberculosis* [19]), or the generation time (delay between primary and secondary infection) short (e.g. *Streptococcus pneumoniae* [20]). In these cases, transmission pairs cannot be accurately identified by genetic data alone, resulting in an overall poorly resolved transmission tree. The informativeness of genetic sequence data is also limited by complex evolutionary behavior. Didelot *et al*. demonstrated that realistic genetic models accounting for within-host diversity, in which several strains coexist inside a host and can be transmitted and sampled, place significant uncertainty around ancestry allocation even when genetic diversity across the outbreak is high, as multiple transmission scenarios are consistent with the genetic data [15]. Pathogens displaying significant within-host diversity include those with long periods of carriage (e.g. *Staphylococcus aureus* [21]) or a propensity for super-infections (*Streptococcus pneumoniae* [22]). WGS is also uninformative of the direction of transmission between donor-recipient pairs if multiple sequences per host are not available [23]. Finally, WGS will generally not be available for all infected individuals, especially in resource poor settings. In the 2014 Ebola outbreak in West Africa, for example, sequences were collected in only 5% of cases [24]. Genetic data is therefore frequently of limited use in reconstructing transmission trees, and inference methods that rely heavily on it will perform poorly in such circumstances.

Integrating other types of outbreak data is therefore necessary for inferring transmission trees in realistic outbreak situations. A frequently collected and highly informative source of data on likely transmission routes is contact data, an integral component of early outbreak response that describes the network of reported contacts with infected individuals. Contact data provided most of the information used to reconstruct transmission chains during Severe Acute Respiratory Syndrome (SARS) [25], Middle East Respiratory Syndrome (MERS) [26] and Ebola [1,27,28] epidemics, and is routinely collected in outbreaks of HIV [29] and Tuberculosis [30]. Contact data can be classified as ‘*exposure*’ data and or ‘*contact tracing*’ data. *Exposure* data describes contacts between a given case and their potential infectors and is an intrinsic part of case definition in diseases with person-to-person transmission. *Contact tracing* data describes contacts between confirmed/probable cases and individuals they could have infected: it is used for active case discovery and rapid isolation and is an integral part of containment strategy. Importantly, both types of contact data potentially contain information on the topology of the transmission tree.

Here, we introduce a model which exploits contact data alongside dates of symptom onset, information on the incubation period (delay between infection and symptom onset) and generation time, and pathogen WGS to reconstruct transmission chains. Our methodology extends the *outbreaker* model introduced by Jombart *et al*. [6] with a contact model that accounts for partial sampling and the presence of non-infectious contacts between cases. As an improvement over other approaches, the integration of a full contact model reduces the reliance on high quality genetic data for accurate inference. We evaluate the performance of this new model and compare the value of the different types of data for inferring who infects whom, using a variety of simulated outbreak scenarios. We then apply our approach to the early stages of the 2003 SARS outbreak in Singapore, integrating the available data on contact structures and genome sequences in a single statistical framework for the first time. The inference tool presented in this study is freely available as the package *outbreaker2* for the R software [31].

## Results

### Algorithm performance on simulated outbreaks

We tested our new model on simulated outbreaks of two pathogens with well-defined epidemiological and evolutionary parameters, namely EBOV and SARS-CoV [27,32]. As SARS-CoV WGS generally contain greater genetic diversity between transmission pairs and are therefore more informative of transmission events than Ebola WGS [17], we describe contrasting outbreak settings where the added value of incorporating contact data may vary. Outbreaks were simulated using empirical estimates of the generation time distribution, the incubation period distribution and the basic reproduction number R_0_ (i.e. the average number of secondary infections caused by an index case in a fully susceptible population [33]). To reflect observed heterogeneities in infectiousness, outbreaks were simulated under strong super-spreading tendencies, where a small number of individuals account for a high number of cases [2,25,34]. Genetic sequence data was simulated using estimates of the genome length and genome wide mutation rate.

To describe contact tracing efforts in various outbreak scenarios, contact data was simulated using two parameters (for a full description of the model, see Methods). Briefly, the probability of a contact being reported is described by ε, the contact reporting coverage. Non-infectious mixing between cases that obscures the topology of the underlying transmission network is described using the non-infectious contact probability *λ*, defined as the probability of contact occurring between two sampled cases that do not constitute a transmission pair. A useful corollary term to *λ* is the expected number of non-infectious contacts per person, *ψ*, as this accounts for the size of the outbreak and describes the amount of non-infectious mixing in terms of numbers of contacts.

We investigated the effect of the coverage of contact tracing efforts and the probability of non-infectious contact on our ability to reconstruct transmission trees using using a grid of values for *ε* and *ψ*. The informativeness of different types of outbreak data was determined by reconstructing each outbreak four times, using combinations of times of sampling (T), contact tracing data (C) and genetic sequence data (G): T, TC, TG and TCG. For an example of a simulated transmission network, contact network and reconstructed transmission tree, see S1 Fig.

**Fig 1:**
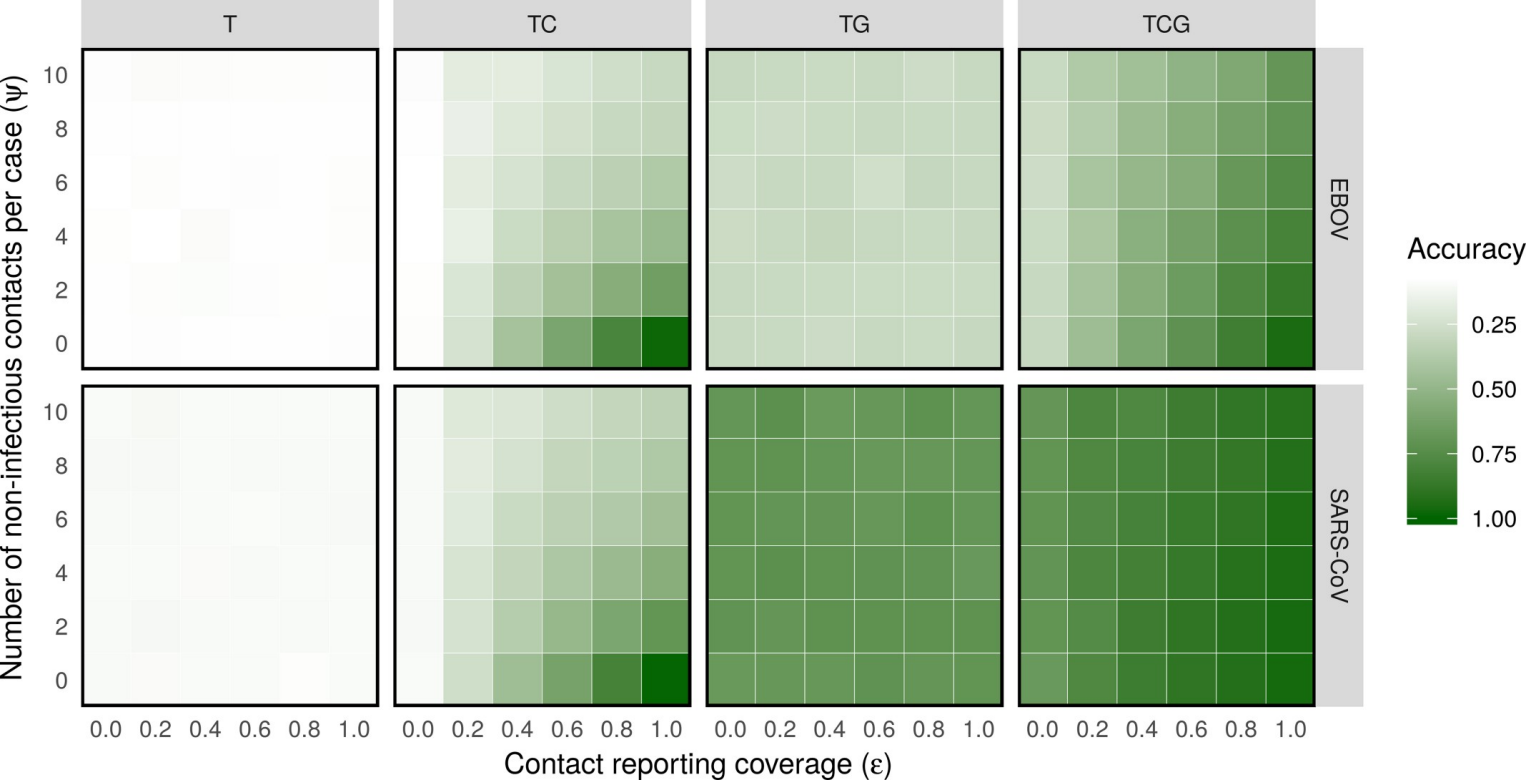
Accuracy of outbreak reconstruction using different types of outbreak data. 100 outbreaks were simulated and reconstructed at each grid point, using different values for the contact reporting coverage *ε* and number of non-infectious contacts per case *ψ*. Each outbreak was reconstructed four times, using different combinations of times of sampling (T), contact tracing data (C) and genetic data (G). The color of a grid point represents the average accuracy of outbreak reconstruction.

Transmission tree reconstruction was essentially impossible using only times of sampling, with on average only 9% and 10% of infectors correctly identified in the consensus transmission tree for EBOV and SARs-CoV outbreaks, respectively (Fig 1). Statistical confidence in ancestry allocation as defined by the average Shannon entropy of the posterior distribution of potential infectors for each case, for which a value of 0 indicates complete posterior support for a given ancestry and higher values indicates lower statistical confidence, was also low (S2 Fig). Including genetic data improved both the accuracy of inference and the statistical confidence in these assignments. However, even in the idealized scenario of error free sequencing and WGS for all cases, this data was insufficient for complete outbreak reconstruction under our genetic likelihood, with on average only 29% and 70% of transmission pairs correctly inferred in in EBOV and SARS-CoV outbreaks, respectively.

Incorporating contact tracing data using our new contact model improved the accuracy of transmission tree reconstruction across all simulations, with the magnitude of improvement dependent on the values of *ε* and *ψ* (Fig 1). Unsurprisingly, accuracy of inferred ancestries increased with coverage *ε*, as a greater number infectious contacts were reported, and decreased with the number of non-infectious contacts *ψ*, as these reduced the proportion of contacts informative of transmission events. In the idealized scenario of complete contact tracing coverage and zero non-infectious contacts, outbreaks were reconstructed with near perfect accuracy, even in the absence of genetic data, with the few incorrectly assigned ancestries attributable to misinformative sampling times. Encouragingly, improvements in accuracy persisted in more realistic contact tracing scenarios with partial coverage and large numbers of non-infectious contacts. For example, consider the contact tracing scenario with only 60% coverage and on average two non-infectious contacts per person. When adding this data to the purely temporal *outbreaker* model, the accuracy in reconstructing EBOV outbreaks increased from 9% to 44%. Though more than half of ancestries remained incorrectly assigned, outbreaks were in fact reconstructed with greater accuracy than when using WGS from Ebola cases, for which accuracy was only 28%.

**Fig 2:**
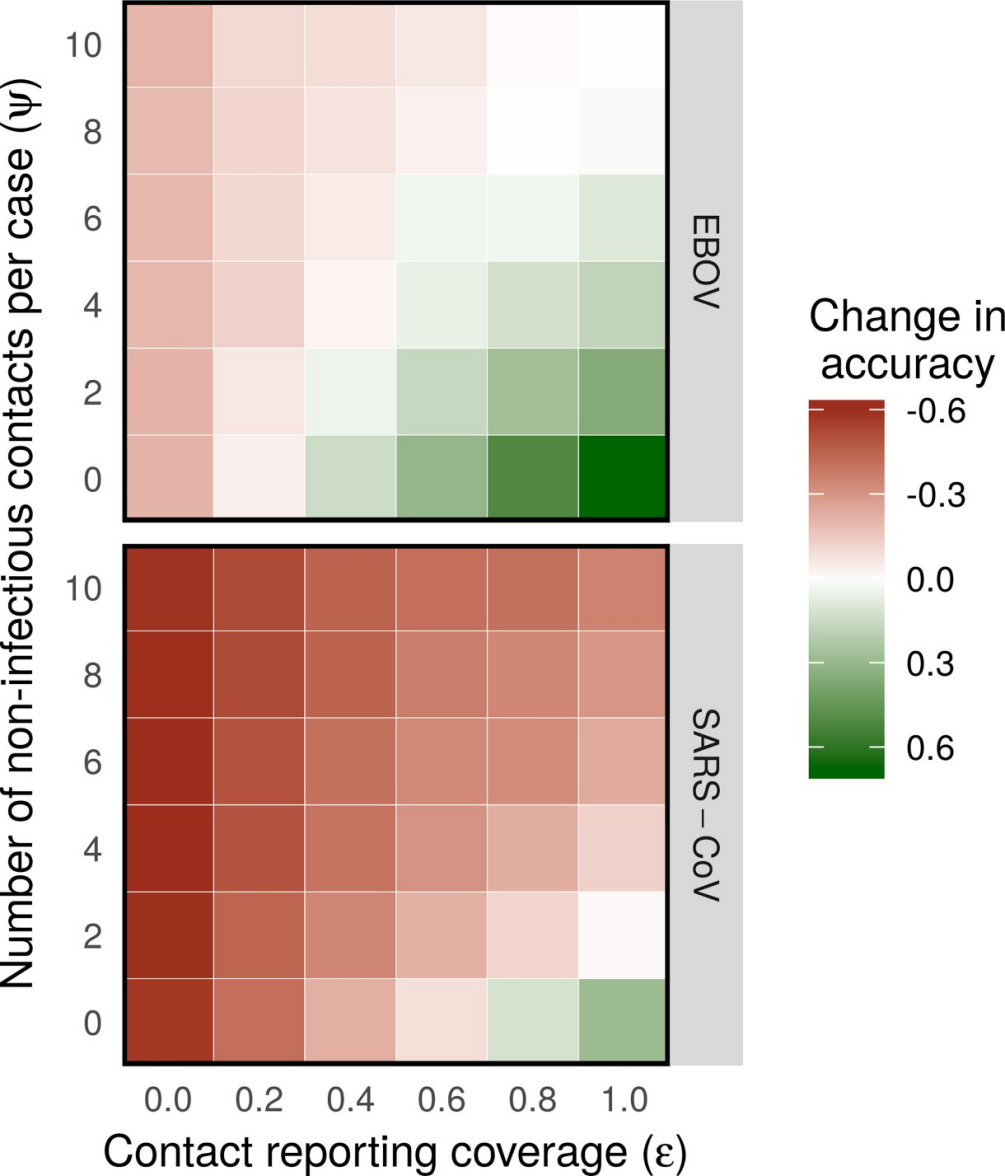
Informativeness of contact data relative to fully sampled genetic data. 100 outbreaks were simulated and reconstructed at each grid point, using different values for the contact reporting coverage *ε* and number of non-infectious contacts *ψ*. The color of a grid point represents the difference between accuracy of outbreak reconstruction using times of sampling and contact tracing data and using times of sampling and genetic data.

When comparing the informativeness of contact data and genetic data across all simulations, we found that information on contact structures was frequently equally or more informative than fully sampled and error-free genetic sequence data, even under limitations of partial coverage and significant levels of non-infectious contact (Fig 2). For example, contact data with only 40% coverage and 4 non-infectious contacts per person was as informative as fully sampled Ebola genetic data. Similarly, if the reporting coverage was 100%, contact data was as informative as Ebola WGS even when individuals reported 10 non-infectious contacts with other cases on average, meaning that only 17% of reported contacts represented true transmission pairs. Though contact data was generally less informative than SARS-CoV WGS in most scenarios, it still provided comparable increases in accuracy when coverage was high (*ε* > 0.6) and contact of non-infectious contact low (*ψ* < 2).

As expected, accuracy of outbreak reconstruction was highest when using contact, temporal and genetic data at the same time. Notably, contact data was able to correct a significant portion of ancestries falsely assigned using only temporal data and WGS. For example, incorporating contact data with 80% coverage and 2 non-infectious contacts per person lead to an increase in average accuracy of outbreak reconstruction from 28% to 79% for EBOV outbreaks (Fig 1). Contact data therefore contained significant additional information on likely transmission routes not available from pathogen WGS, which was successfully integrated in our inference framework.

In addition to the transmission tree itself, we inferred the model parameters *ε* and *λ* under uninformative priors and observed accurate estimates of the simulated values for both EBOV and SARS-CoV outbreaks (S3 and S4 Figs). When using temporal and contact data, the mean posterior estimates of *ε* and *λ* across 100 outbreaks were generally distributed around the true simulated value, and with low variance especially when the coverage *ε* was high. Only when *λ* was high were the estimates slightly off-centered from the true value. Including genetic data improved parameter inference across all scenarios, resulting in correctly centered estimates with a reduced variance. *ε* and *λ* are therefore identifiable in our contact likelihood and generally well estimated by our inference framework, allowing appropriate probabilistic weighting of contact data in the allocation of ancestries.

### 2003 SARS outbreak in Singapore

We applied our method to the early stages of the 2003 SARS outbreak in Singapore, for which dates of symptom onset, whole genome sequences and contact information were collected for the first 13 cases [35,36]. Previous attempts to infer the transmission tree from these data either reconstructed probable lineages by manual inspection [35,36], or entirely discarded information on the six reported contacts between cases [6,37], even though they were all thought to be epidemiologically significant [36].

**Fig 3:**
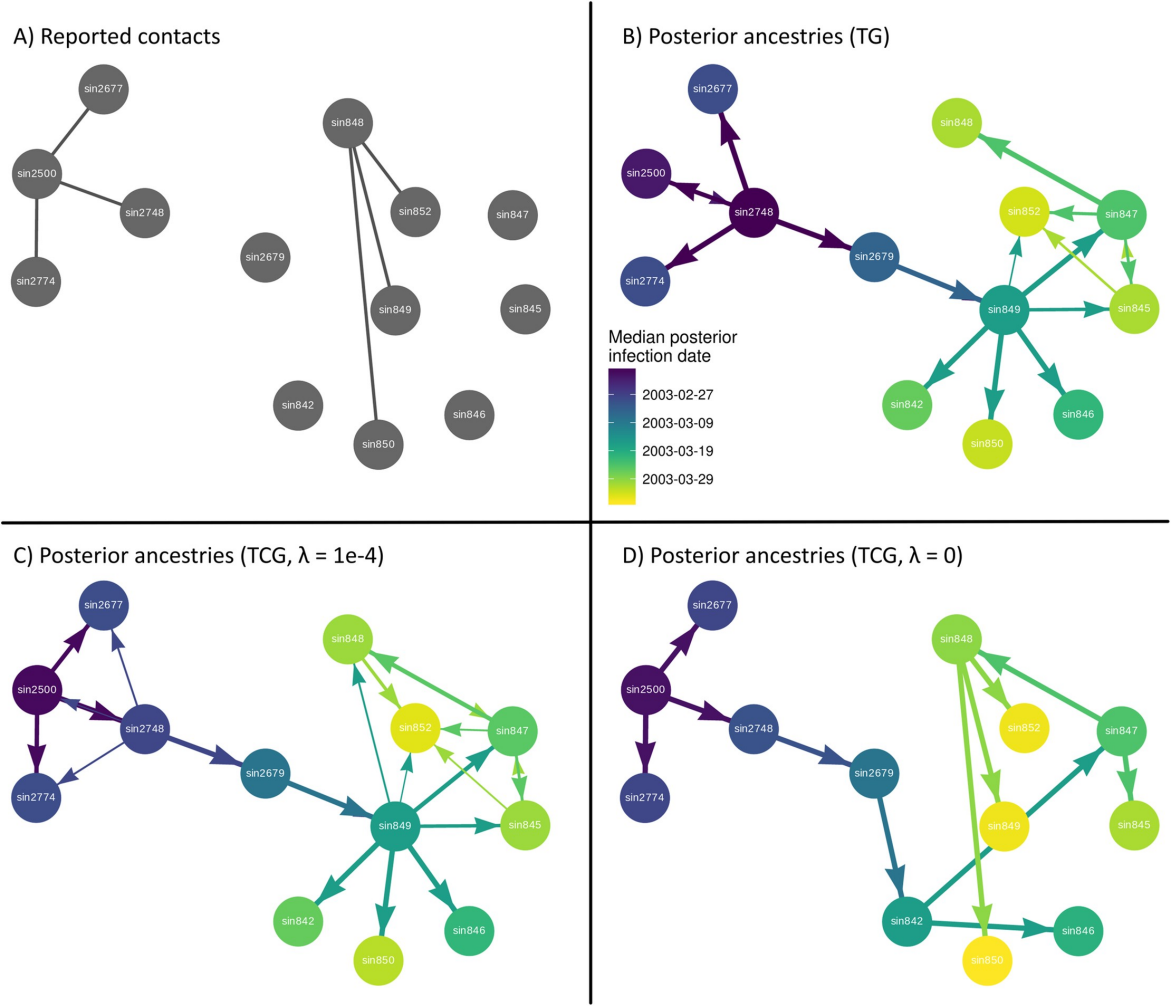
Reconstruction of the 2003 SARS outbreak in Singapore. **A)** Circles represent individual cases, and edges the epidemiological contacts reported between them. **B)** The outbreak was reconstructed using temporal and genetic data. Arrows represent posterior ancestries between cases, scaled in width by the posterior frequency of that ancestry. Ancestries with a minimum posterior frequency of 0.01 were included. The color of a node corresponds to the median posterior infection time of that case. **C)** The outbreak was reconstructed using temporal, contact and genetic data, and the non-infectious contact probability *λ* fixed at a value of 1e-4. **D)** The outbreak was reconstructed using temporal, contact and genetic data, and the non-infectious contact probability *λ* fixed at a value of 0.

Using *outbreaker2*, we were able to infer the range of transmission histories consistent with the temporal, genomic and contact data in a probabilistic manner. We analyzed the outbreak several times using different settings; with and without contact data and using different priors on *λ* (Fig 3). Under the assumption that the reported contacts were very likely to be epidemiologically relevant, by fixing the non-transmission contact rate *λ* at 1e-4, contact data significantly changed the posterior distribution of ancestries (Fig 3B and 3C). As expected under these assumptions, transmission links in line with reported contacts were better supported. For example, the most likely infector of cases *sin2677* and *sin2774* was *sin2500* when including contact data (Fig 3C), instead of *sin2748* in the default analysis (Fig 3B). Even though these transmission events were less likely under the genetic likelihood, as they implied the accumulation of 2 and 3 mutations, respectively, rather than 1 and 2 mutations, these ancestries were supported by the contact data and were therefore credible under our model. Importantly, the original transmission pathway inferred in the absence of genetic data (*sin2748* infecting *sin2677* and *sin2774*) also remained plausible. Further novel infection routes supported by contact data were *sin849* infecting *sin848*, and *sin848* infecting *sin852*.

However, not all ancestries supported by contact data received significant posterior support. Even though *sin849* was in contact with and therefore a likely infector of *sin848*, *sin847* remained the consensus ancestor of *sin848* with 78% posterior support, as it is separated from *sin848* by only 1 mutation, which is far more favorable under the genetic likelihood compared to the 7 mutations separating *sin849* and *sin848*. Furthermore, though *sin850* and *sin848* had a reported contact, an infectious relationship between the two received no posterior support due to the large number of mutations (10) separating the two. Therefore, while the contact model generally provided support for transmission histories in line with epidemiological observations of contacts, each ancestry allocation was the result of weighing the evidence provided by all three, potentially conflicting, data sources.

Interestingly, incorporating contact data in our analysis affected ancestry allocations not directly referenced in the contact network. For example, *sin848* was suggested as a novel infector of *sin847* with 22% posterior support, though these cases are not linked by a reported contact. This is explained by a change in the inferred infection times (S5 Fig). *sin848* infecting *sin852*, as suggested by the contact data, resulted in an earlier inferred infection time for *sin848*, which in turn made it a plausible infector of *sin847*. A similar change in the inferred infection times of *sin2500* and *sin2748*, driven by the contact data, reversed the directionality of their consensus infectious relationship, even though this directionality was not provided in the contact data. Incorporating the contact model alongside the genetic and temporal model therefore allowed for high level interactions, beyond simply providing support for ancestries indicated in the contact data.

We also analyzed the dataset using a weaker prior on *λ* (Beta(1, 10)) and an uninformative prior. However, the resulting posterior ancestries were essentially identical to those inferred in the absence of contact data (S6 Fig).

We then reconstructed the outbreak under the assumption that all reported contacts necessarily occurred between direct transmission pairs by fixing *λ* at a value of 0 (Fig 3D). The posterior distribution of transmission networks therefore spanned the contact network, with 6 of the 12 ancestries remaining fixed. This rigid topology of plausible transmission networks resulted in low variance among the remaining ancestries, producing essentially a single posterior tree. Notably, this analysis proposed several new ancestries (*sin2679* to *sin842*, *sin842* to *sin847* and *sin848 to sin850*) rejected with a *λ* value of 1e-4 and had a substantially lower average log-likelihood (-647.4 compared to -579.2). Therefore, while the assumption that *λ* was 0 may have been valid, this approach forced the algorithm to accept ancestries highly unlikely under the genetic and temporal likelihoods, thereby preventing a meaningful integration of different data sources.

## Discussion

The methodology described here represents, to our knowledge, the first outbreak reconstruction framework integrating contact data alongside the timing of symptom onset, reporting rates and pathogen WGS data. Using simulations, we have shown how contact data can improve epidemiological inference across a range of outbreak settings, including incomplete contact tracing coverage, significant amounts of non-infectious contact and strong super-spreading tendencies. By integrating contact data in the analysis of early stages of the 2003 Singaporean SARS outbreak for the first time, we have illustrated how our approach can work in a realistic outbreak scenario and provide a probabilistic description of plausible transmission routes in the face of conflicting outbreak data. The general applicability of our model, in addition to being implemented in a freely available and well-documented software package, makes *outbreaker2* useful to a broad epidemiological audience.

Our work reduces the reliance of outbreak reconstruction tools on WGS data. This is significant when considering that genetic diversity in many pathogens arises too slowly to resolve a significant portion of transmission pairs by genetic means [17], and that within-host genetic diversity of other pathogens hinders accurate transmission tree reconstruction from genetic data [38]. Furthermore, sequencing pathogen genomes from enough cases in an outbreak to resolve individual transmission events is frequently unrealistic in the face of logistical and financial limitations [24]. In contrast, contact tracing is routinely conducted during outbreak response, and therefore provides a valuable additional window of information on transmission events without placing an additional burden on field epidemiologists. Indeed, given the simulation model and likelihoods used for inference, our work suggests that even incomplete contact tracing data may be more informative than fully sampled, error-free genetic sequence data of some pathogens.

Methodologically, our contact model differs from previous methods for relating contact data to epidemiological processes, with several advantages [39–41]. Soetens *et al*. estimate effective reproduction numbers by assigning transmission links on the basis of contact data, while accounting for right censoring of case counts [39]. However, they assume complete sampling of contacts and cases, and automatically designate confirmed cases with a known contact as transmission pairs. This is equivalent to fixing λ at a value of 0 in our model, which our analysis of the SARS dataset has shown is unsuitable for integrating other types of data in a meaningful manner. Similarly, Hens *et*. *al*. restrict transmission pairs to those supported by reported contacts [40], thereby mis-assigning ancestries if contacts are only partially reported.

Jewell and Roberts establish a more statistically rigorous approach for epidemiological inference from contact data by explicitly modelling the contact process that drives the infectious process in an SINR compartmental framework [41]. Such a mechanistic model natively relates epidemiological processes to a set of observed contact data and has the advantage of potentially accommodating complex contact structures caused by non-random mixing in the future. However, a prospective model of this sort is considerably more complex to develop in a statistically tractable manner and has necessitated the assumption of a single index case, whereas multiple infectious introductions are easily accounted for in our contact likelihood. Furthermore, their approach does not explicitly model under-reporting of contacts, and therefore does not allow valuable prior information on the coverage of the contact tracing effort to inform the analysis. Our approach is therefore applicable to a wider range of realistic outbreak settings.

Incorporating this contact model alongside a temporal and genetic model represents an improvement over previous, ad-hoc methods to data integration, which generally use contact data to exclude transmission links and then explore the remaining transmission tree space using other data [42,43]. By modelling contact tracing as a probabilistic process in a Bayesian framework, information on the contact tracing effort can also be embedded in the prior to improve the inferential process and more explicitly describe the assumptions underlying it. For example, if most contacts in an outbreak are expected to have been reported, the prior on the contact reporting coverage *ε* can be shifted to provide greater support for higher values, reducing support for ancestries that lack a contact. *ε* could even be fixed at a value of 1, meaning a reported contact is *required* for a given transmission pair to be inferred, given the assumption that every contact has been reported. Similarly, as shown for the 2003 SARS outbreak, an informative prior on the non-infectious contact probability *λ* should generally be used. As most contact tracing efforts are conducted under the belief that non-transmission pairs experience contacts with significantly lower probability than transmission pairs, the prior on *λ* should provide support for lower values, in turn placing greater weight on reported contacts when assigning ancestries.

Our method also allows conflicting data to be treated in a systematic manner, as demonstrated by the analysis of the the 2003 SARS outbreak, where several ancestries were supported by contact data yet separated by an implausibly large number of mutations. In contrast to existing tools [6,35], *outbreaker2* can evaluate these inconsistencies and determine the distribution of likely transmission trees under multiple data types. While not necessarily improving the accuracy of the inferred transmission tree, our approach better captures the uncertainty around these ancestry assignments given the available data.

However, it is important to note both the intrinsic informational limitations of contact data as well as the methodological limitations of the work presented here. Contact tracing constitutes a significant logistical challenge, as most if not at all infected individuals must be followed up, and suspected cases monitored past the upper end of the incubation period distribution [44–46]. The coverage of contact tracing efforts conducted in low resource settings may therefore be low [47], and consequently poorly informative of the transmission network (Fig 1). Even if a significant proportion of contacts are reported, a high degree of mixing between cases can obscure the topology of the underlying transmission network, for example within hospital wards or classrooms. Contact data alone will therefore not always suffice for complete reconstruction of an outbreak. Nevertheless, the framework presented here allows even minimally informative contact data to be incorporated into transmission tree inference alongside other available data.

Furthermore, the use of strong priors on *ε* and *λ* may be required to ensure adequate weighting of contact data, especially in the face of conflicting genetic data as shown in the analysis of the 2003 SARS outbreak. While our framework forces an explicit description of these assumptions, the sensitivity of the algorithm outputs to the prior distributions should be noted and explored adequately.

Our model of epidemiological contacts also makes a number of simplifications, some of which could be improved upon in future work. As the contacts are undated, the model does not consider that they are only indicative of transmission events if they occur during the infectious period of the infector, potentially resulting in overconfident ancestry assignments if contacts frequently occur outside this time period. However, as epidemiologists generally only record meaningful contacts occurring within likely windows of infection, the assumption that recorded contacts represent epidemiologically plausible transmission pairs appears reasonable. As currently implemented, our model also does not account for different weights between contacts, which could be useful for example to stratify different types of sexual intercourse by their risk of HIV transmission [48], or TB contacts by their duration of contact (e.g. household vs. casual). However, it could be easily extended to do so by using separate parameters for the reporting coverage (e.g. *ε*_1_, *ε*_2_, *ε*_3_) and non-infectious contact probability (e.g. *λ*_1_, *λ*_2_, *λ*_3_) of each type of contact. Furthermore, the contact model is undirected and treats exposure data and contact tracing data equally, resulting in a loss of information about the potential directionality of the infectious interaction which must instead be inferred from other data. Directionality could be incorporated with relative ease by treating reported contacts as asymmetric (individual *i* contacting individual *j* is distinct from *j* contacting *i*) and relating this to the infector-infectee relationship in the putative transmission tree (*I* infecting *j* is distinct from *j* infecting *I*). However, the current model generally inferred directionality successfully from temporal data simulated under realistic delay distributions (Fig 1).

It should also be noted that the use of fixed generation time and incubation period distributions is poorly suited to epidemic scenarios with highly connected contact networks, for which hazard-based approaches are more suitable [49,50]. However, as demonstrated in Fig 1, contact data is only informative when the contact network itself is fairly sparse (i.e. *λ* is low). The assumption of fixed generation time and incubation period distributions is therefore suitable for the use cases of our contact model [10,13,51].

Finally, the assumptions underlying the pairwise genetic model should be considered when using *outbreaker2*. The likelihoods of pairwise genetic distances are treated as independent, when in fact they are dependent on the underlying infectious relationships between cases (e.g. the genetic relatedness of case A and its infector B is dependent on the infector of B). Similarly, by considering only genetic distances, our method disregards histories of shared mutations between genomes. These assumptions can result in loss of information and potential misinterpretation of genetic signals, especially when evolutionary histories are complex [38]. In such cases, character-based, phylogenetic models should be considered [10,11].

In conclusion, the work presented here provides a simple yet flexible methodology for integrating contact data with genetic and temporal data in the inference of transmission trees. By allowing contact data to complement and/or substitute genetic data as the primary source of information on infectious relationships between individuals, our work increases both the scope and accuracy of methodologies for outbreak reconstruction.

## Methods

### Outbreaker model

Our work is an extension of the *outbreaker* model developed by Jombart et al. [16], re-written in a manner to be more extensible This model considers, for each case *I* (*i* = 1, …,N), the probability of a proposed transmission history given the time of symptom onset *t*_*i*_ and a pathogen genetic sequence *s*_*i*_ (Table 1). Assumptions on the temporal relationship between transmission pairs are given by the generation time distribution *w*, defined as the distribution of delays between infection of a primary and secondary case, and the incubation period distribution *f*, defined as the distribution of intervals between infection and symptom onset of a case. *w* and *f* are assumed to be known, and not estimated during the inference process.

**Table 1:** Notation of outbreaker model [6].

Symbol	Type	Description
***i***	Data	Index of cases
***N***	Data	Number of cases in the sample
***s***_***i***_	Data	Sequence of case *i*
***t***_***i***_	Data	Collection date of *s*_*i*_
***c***_***i*,*j***_	Data	Contact status between case *i* and case *j*
***w***	Function	Generation time distribution
***f***	Function	Incubation period distribution
***d(s***_***i***_**,*s***_***j***_***)***	Function	Number of mutations between *s*_*i*_ and *s*_*j*_
***l(s***_***i***_**,*s***_***j***_***)***	Function	Number of comparable nucleotide positions between *s*_*i*_ and *s*_*j*_
***α***_***i***_	Augmented data	Index of the most recent sampled ancestor of case *i*
***κ***_***i***_	Augmented data	Number of generations between *α*_*i*_ and *i*
***T***_***i***_^***inf***^	Augmented data	Date of infection of *i*
***μ***	Parameter	Mutation rate, per site and per generation of infection
***π***	Parameter	Proportion of cases sampled in the outbreak
***ε***	Parameter	Proportion of contacts reported
***λ***	Parameter	Probability of non-infectious contact between cases
**η**	Parameter	Probability of contact between transmission pairs
**ζ**	Parameter	Probability of false-positive reporting a contact

The unobserved transmission events are modelled using augmented data; case *i* is infected at time *T*_*i*_^*inf*^, and its most recent sampled ancestor denoted *α*_*i*_. To allow for unobserved cases, the number of generations separating *i* and *α*_*i*_ is explicitly modelled and denoted *κ*_*i*_ (*κ*_*i*_ ≥ 1). The proportion of cases that have been sampled is defined by the parameter π and is inferred as part of the estimation procedure. The other estimated parameter is the mutation rate *μ*, measured per site per generation of infection.

This model is embedded in a Bayesian framework. Denoting *D* the observed data, *A* the augmented data and θ the model parameters, the joint posterior distribution of parameters and augmented data is defined as:

$$P(A,\theta |D)=\frac{P(D,A|\theta )P(\theta )}{P(D)}$$

The first term describes the likelihood of the data, the second term the joint prior (for a complete description of both, see Jombart *et al*. [6]). Briefly, the likelihood is computed as a product of case-specific terms, and can be decomposed into a genetic likelihood Ω^1^, a temporal likelihood Ω^2^ and a reporting likelihood Ω^3^.

The genetic likelihood describes, for a given case *i*, the probability of observing the genetic distance between sequence *s*_*i*_ and that of its most recent sampled ancestor *s*_*αi*_, given the proposed ancestries and parameters:

$$\Omega_{i}^{1}=p(s_{i}|\alpha_{i},s_{\alpha_{i}},\kappa_{i},\mu )$$

and is defined as:

$${\left(\kappa_{i}\mu \right)}^{d(s_{i},s_{\alpha_{i}})}{\left(1-\kappa_{i}\mu \right)}^{l(s_{i},s_{\alpha_{i}})-d(s_{i},s_{\alpha_{i}})}$$

This calculates the probability of d(s_i_,s_j_) mutation events occurring at the observed nucleotide positions and no mutations occurring at the remaining positions, while summing over the *κ*_*i*_ generations in which the mutations could have occurred. For a full derivation of this likelihood, see S1 Text. The temporal likelihood describes the probability of observing the time of symptom onset and proposed time of infection:

$$\Omega_{i}^{2}=p(t_{i}|T_{i}^{inf})p(T_{i}^{inf}|\alpha_{i},T_{\alpha_{i}}^{inf},\kappa_{i})$$

and is calculated as:

$$f(t_{i}-T_{i}^{inf})w^{\kappa_{i}}(T_{i}^{inf}-T_{\alpha_{i}}^{inf})$$

*w*^*κ*^ = w*w*…*w, where * is the convolution operator and is applied *k* times. The first term describes the probability of the imputed time of infection under the incubation period distribution. The second term describes the probability of observing the delay between infection times of the case and its most recent sampled ancestor under the generation time distribution, over the imputed number of generations. The reporting likelihood describes the probability of unobserved intermediate cases:

$$\Omega_{i}^{3}=p(\kappa_{i}|\pi )$$

and is calculated as:

$$NB(1|\kappa_{i}-1,\pi )$$

where NB is the probability mass function of the negative binomial distribution, and describes the probability of not observing *κ*_*i*_—1 cases given a probability of observation of *π*.

### Contact likelihood

**Fig 4:**
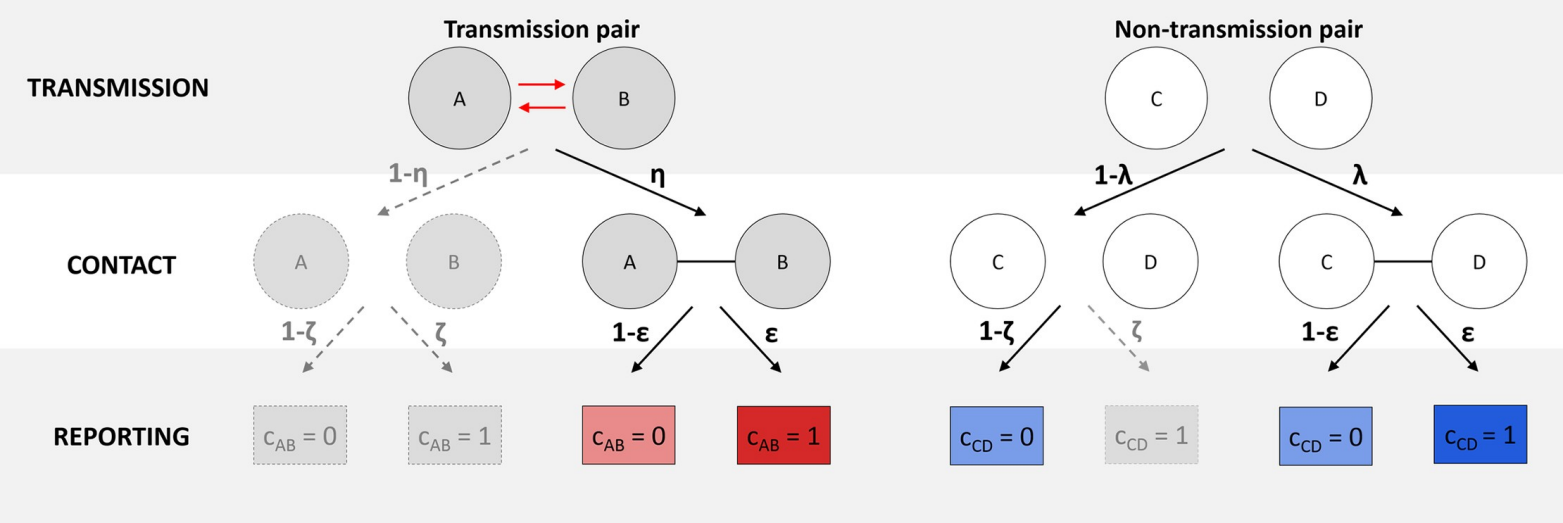
Modelling contact data from transmission trees. Circles represent sampled, infected individuals. *c*_*i*,*j*_ represents the contact status between cases *i* and *j*, with 1 indicating a reported contact and 0 the absence of a reported contact. Transmission pairs and non-transmission pairs experience contact with probabilities *η* and *λ*, respectively. These contacts are reported with probability *ε*. False positive reporting of contacts that have not occurred occurs with probability ζ. In the simplified model implemented in *outbreaker2*, as indicated by colored shading and solid outlines, *η* is assumed to be 1 and *ζ* assumed to be 0.

To integrate contact data into *outbreaker*, we developed a method for modelling contact data from transmission trees (Fig 4). The model considers undated, undirected, binary contact data, such that the contact status *c*_*i*,*j*_ is set to 1 if contact is reported between individuals *i* and *j* and set to 0 otherwise. The model is hierarchical and describes two processes: the occurrence of contacts and the reporting of contacts. Transmission pairs experience contact with probability *η*. This formulation accounts for the possibility of transmission occurring without direct contact, for example by indirect environmental contamination as is observed with *Clostridium difficile* [52]. Sampled, infected individuals that do not constitute a transmission pair experience contact with probability *λ*, the non-infectious contact probability. Contacts that have occurred, either between transmission pairs or non-transmission pairs, are then reported with probability *ε*, the contact reporting coverage. Contacts that have not occurred are reported with probability *ζ*, the false positive reporting rate.

We make two assumptions to simplify this model, which can be relaxed in future work if necessary. Firstly, we assume that direct contact is necessary for transmission and set *η* to 1. Furthermore, we assume that false reporting of contacts that have not occurred is negligible and set *ζ* to zero. This model allows us to define a contact likelihood Ω^4^, describing the probability of observing the contact data *C* (a symmetrical, binary, NxN adjacency matrix with zeros on its diagonal) given a proposed transmission tree and parameters *ε* and *λ*. Formally, for individual *i*:

$$\Omega_{i}^{4}=\prod_{i=1,j\neq i}^{N}p(c_{i,j}|\alpha_{i},\kappa_{i},\epsilon ,\kappa )$$

Using the contact model described in Fig 4 and the simplifying assumptions made above:

$$p(c_{i,j}=1|\alpha_{i}=j,\kappa_{i}=1)=\epsilon$$

$$p(c_{i,j}=0|\alpha_{i}=j,\kappa_{i}=1)=1-\epsilon$$

$$p(c_{i,j}=1|\alpha_{i}\neq j)=p(c_{i,j}=1|\alpha_{i}=j,\kappa_{i}>1)=\lambda \epsilon$$

$$p(c_{i,j}=0|\alpha_{i}\neq j)=p(c_{i,j}=0|\alpha_{i}=j,\kappa_{i}>1)=(1-\lambda )+\lambda (1-\epsilon )$$

For a mathematical description of the unsimplified model, see S2 Text. The updated joint posterior distribution is therefore proportional to the product of the four likelihood terms and the joint prior:

$$P(A,\theta |D)\propto p(\alpha ,\mu ,\pi ,\epsilon ,\lambda )\prod_{i=1}^{N}\Omega_{i}^{1}\Omega_{i}^{2}\Omega_{i}^{3}\Omega_{i}^{4}$$

### Prior distributions

The prior distributions are assumed independent, such that:

p(α,μ,π,ϵ,λ)=p(α)p(μ)p(π)p(ϵ)p(λ)

The prior on ancestries p(*α*) is uniform, and the prior on the mutation rate *μ* exponentially distributed. *π*, *ε* and *λ* represent probabilities and are assigned Beta distributed priors with user-defined parameters, to allow flexible specification of previous knowledge on the sampling coverage, contact reporting coverage and non-infectious contact probability.

### Simulation scenarios

Transmission trees and genetic sequence evolution were simulated using the *simOutbreak* function from the R package *outbreaker*. To describe heterogeneities in infectiousness within a population, well-documented in both EBOV [34] and SARS-CoV [25] outbreaks, and capture consequent ‘superspreading’ events, in which a small portion of the population accounts for a large number of infections, we described the ‘individual reproductive number’ R_*i*_, a variable describing the expected number of secondary cases caused by a particular infected individual [2]. Following previous studies by Lloyd-Smith *et al*. [2] and Grassly and Fraser [53], we assumed R_*i*_ to be Gamma distributed with a mean of R_0_ and a dispersion parameter *k*, with lower values of *k* indicating greater heterogeneity in infectiousness. The resulting offspring distribution is a negative binomial [2].

Estimates of the generation time distribution, R_0_, mutation rate and genome length were taken from a literature review described by Campbell *et al*. [17] Estimates of the incubation period distribution and dispersion parameter of R_*i*_ were drawn from the literature (Table 2). Generation time distributions and incubation period distributions were described by discretized gamma distributions, generated using the function *DiscrSI* from the R package *EpiEstim* [54].

**Table 2:** Epidemiological and genetic parameters for EBOV and SARS-CoV.

Parameter	EBOV	SARS-CoV
Mean generation time in days (SD)	14.4 (8.9) [1,55,56]	8.7 (3.6) [57–59]
Mean incubation period (SD)	9.1 (7.3) [55]	6.4 (4.1) [60]
Mean R_*i*_ (dispersion)	1.8 (0.18) [34,55]	2.7 (0.16) [2,32]
Mutation rate (per site per day)	0.31 x 10^−5^ [61–63]	1.14 x 10^−5^ [36,64,65]
Genome length (bases)	18958 [61,66]	29714 [35,67]

Contact data was simulated from transmission trees using the model described in Fig 3, using a grid of values for the reporting coverage (*ε* ∊ [0, 1]) and the number of non-infectious contacts per person (*ψ* ∊ [0, 10], *λ* ∊ [0, 0.18]). For a mathematical description of the relationship between *ψ* and *λ*, see S3 Text. At each grid point, 100 outbreaks were simulated, with a single initial infection in a susceptible population of 200 individuals. Simulations were run for 100 days, or until no more infectious individuals remained. The first 60 ancestries of each outbreak were reconstructed four times using the R package *outbreaker2*, using combinations of times of symptom onset (T), contact data (C) and WGS (G): T, TC, TG and TCG. For each analysis, one MCMC chain was run for 10,000 iterations with a thinning frequency of 1/50 and a burn-in of 1,000 iterations. The prior distributions used for *ε* and *λ* were uninformative (Beta(1,1)), and default priors used otherwise.

### Quantifying accuracy and statistical confidence

The accuracy of outbreak reconstruction was defined as the proportion of correctly assigned ancestries in the consensus transmission tree, itself defined as the tree with the modal posterior infector for each case. The uncertainty associated with an inferred ancestry was quantified using the Shannon entropy of the frequency of posterior ancestors for each case [68]. Given *K* ancestors of frequency *f*_*K*_ (*k* = 1, …,*K*), the entropy was defined as:

$$-\sum_{k=1}^{K}f_{k}log(f_{k})$$

### Analyzing the 2003 SARS outbreak in Singapore

Thirteen previously published [35,36] and aligned [6] SARS whole genome sequences were obtained for our analysis. Data on epidemiological contacts were described by Vega *et al*. [36]. The same generation time distribution and incubation period distribution used for the analysis of the simulated SARS outbreaks were used (Table 2). As the number of non-transmission contacts was assumed to be low and a total of 6 contacts were reported in an outbreak of 13 cases, the proportion of contacts reported was believed to be about 50%. The prior on ε was therefore chosen as Beta(5, 5). Several priors on the non-transmission contact rate λ were tested; Beta(1, 10), Unif(0, 1), a fixed value of 0 and a fixed value of 1e-4. The priors on the mutation rate μ and proportion of cases sampled π were uninformative. The MCMC chain was run for 1e7 iterations with a thinning frequency of 1/50 and a burn-in of 1,000 iterations.

## Supporting information

S1 Text Derivation of genetic likelihood. (DOCX)

S2 Text Derivation of un-simplified contact model. (DOCX)

S3 Text Derivation of the number of non-infectious contacts per person from the non-infectious contact probability. (DOCX)

S1 Fig Example of simulated transmission tree, contact network and reconstructed transmission tree. **A)** An Ebola-like outbreak of 15 cases was simulated in a susceptible population of 50 susceptible individuals. **B)** A contact network was simulated with a reporting coverage *ε* of 0.8 and a non-infectious contact probability λ of 0.1. Solid lines represent reported contacts; green lines correspond to transmission pairs, red lines to non-transmission pairs. Dashed green lines represent contacts between transmission pairs that were not reported. **C)** The outbreak was reconstructed using temporal and genomic data, and the consensus transmission tree, describing the modal posterior infector for each case, determined. Green lines correspond to correctly inferred ancestries, red lines to incorrectly inferred ancestries. The accuracy of outbreak reconstruction was 46%. **D)** The outbreak was reconstructed using temporal, genomic and contact data, with an accuracy of 94%.
(TIF)

S2 Fig Statistical confidence in ancestry assignment using different types of outbreak data. 100 outbreaks were simulated and reconstructed at each grid point, using different values for the contact reporting coverage *ε* and number of non-infectious contacts per case *ψ*. Each outbreak was reconstructed four times, using different combinations of times of sampling (T), contact tracing data (C) and genetic data (G). The colour of a grid point represents the average entropy of ancestry assignments and is related to the number of plausible infectors of a given case. Lower average entropy indicates greater statistical confidence in the proposed transmission tree.
(EPS)

S3 Fig Parameter estimates of the contact reporting coverage *ε* and non-infectious contact probability λ for simulated EBOV outbreaks. The density plots represent the mean posterior estimates of *ε* and λ across 100 reconstructed outbreaks. The shading represents the data used during the inference process, namely temporal and contact data only (TC), or temporal, contact and genetic data (TCG). The true, simulated value is indicated by a vertical dashed line.
(EPS)

S4 Fig Parameter estimates of the contact reporting coverage *ε* and non-infectious contact probability *λ* for simulated SARS-CoV outbreaks. The density plots represent the mean posterior estimates of *ε* and *λ* across 100 reconstructed outbreaks. The colour of the plot represents the data used during the inference process, namely temporal and contact data only (TC), or temporal, contact and genetic data (TCG). The true, simulated value is indicated by a vertical dashed line.
(EPS)

S5 Fig Infection time estimates for the 2003 SARS outbreak in Singapore. The violin plots indicate the posterior distribution of infection times for the 13 cases in the outbreak. The black dots represent times of symptom onset. The colour of the violin plot indicates the settings used to reconstruct the outbreak, namely using temporal and genetic data only (TG), or temporal, contact and genetic data (TCG). The prior used for the non-transmission contact probability *λ* is indicated in brackets.
(EPS)

S6 Fig Posterior ancestries for the 2003 SARS outbreak in Singapore under different prior distributions for non-infectious contact probability *λ*. Columns represent sampled cases in the outbreak, rows represent potential sampled infectors. The size of each circle represents the posterior frequency of a given infector-infectee pair.
(TIF)
