# Supplementary material for: Bayesian inference of transmission chains using timing of symptoms, pathogen genomes and contact data
Source: PLoS Comput Biol. 2019 Mar 29;15(3):e1006930. doi: 10.1371/journal.pcbi.1006930 (PMC6457559; doi:10.1371/journal.pcbi.1006930)
Supplement: S1 Text — (DOCX) [file pcbi.1006930.s001.docx]

**S1 Text. Derivation of genetic likelihood.**

The genetic likelihood describes, for a given case $i$, the probability of observing the genetic distance $d\left( s_{i},s_{\alpha_{i}} \right)$ between sequence $s_{i}$ and that of its most recent sampled ancestor $s_{\alpha_{i}}$*,* given $i$ and $\alpha_{i}$ are separated by $\kappa_{i}$ generations of infection. The number of comparable nucleotide positions between $s_{i}$ and $s_{\alpha_{i}}$ is denoted $l\left( s_{i},s_{\alpha_{i}} \right)$.

We use an infinite-sites model under the assumption that multiple mutations at the same nucleotide position do not occur. Given a mutation rate $\mu$ per generation of infection, the probability of a mutation occurring at a given nucleotide position over $\kappa_{i}$ generations of infection is $\kappa_{i}\mu$. The probability of observing $d\left( s_{i},s_{\alpha_{i}} \right)$ mutations *at the observed nucleotide positions only* is therefore given by:

$${=\left( \kappa_{i}\mu\right)}^{d\left( s_{i},s_{\alpha_{i}} \right)}\left( 1-\kappa_{i}\mu\right)^{l\left( s_{i},s_{\alpha_{i}} \right)-d\left( s_{i},s_{\alpha_{i}} \right)}$$
