# Supplementary material for: Bayesian inference of transmission chains using timing of symptoms, pathogen genomes and contact data
Source: PLoS Comput Biol. 2019 Mar 29;15(3):e1006930. doi: 10.1371/journal.pcbi.1006930 (PMC6457559; doi:10.1371/journal.pcbi.1006930)
Supplement: S2 Text — (DOCX) [file pcbi.1006930.s002.docx]

**S2 Text. Mathematical description of the unsimplified contact model.**

The contact model considers undated, undirected, binary contact data, such that the contact status *c_i,j_* is set to 1 if contact is reported between individuals *i* and *j*, and set to 0 otherwise. The model is hierarchical and describes two processes: the occurrence of contacts and the reporting of contacts. Transmission pairs experience contact with probability η. Sampled, infected individuals that do not constitute a transmission pair experience contact with probability *λ*. Contacts that have occurred, either between transmission pairs or non-transmission pairs, are then reported with probability *ε*, the contact reporting coverage. Contacts that have not occurred are reported with probability ζ, the false positive reporting rate. Given this model (see Fig 3), the probability of observing the contact data *C* (a symmetrical, binary, NxN adjacency matrix with zeros on its diagonal) given a proposed transmission tree and parameters *η*, *λ*, *ε* and *ζ* is as follows:

$$p(c_{i,j}=1|\alpha_{i}=j,\kappa_{i}=1)=(1-\eta)\zeta+\eta\epsilon$$

$$p(c_{i,j}=0|\alpha_{i}=j,\kappa_{i}=1)=(1-\eta)(1-\zeta)+\eta(1-\epsilon)$$

$$p(c_{i,j}=1|\alpha_{i} \neq j)=p(c_{i,j}=1|\alpha_{i} = j,\kappa_{i}>1)=(1-\lambda)\zeta+\lambda\epsilon$$

$$p(c_{i,j}=0|\alpha_{i} \neq j)=p(c_{i,j}=0|\alpha_{i} = j,\kappa_{i}>1)=(1-\lambda)(1-\zeta)+\lambda(1-\epsilon)$$
