# Supplementary figures and images for: Bayesian inference of transmission chains using timing of symptoms, pathogen genomes and contact data
Source: PLoS Comput Biol. 2019 Mar 29;15(3):e1006930. doi: 10.1371/journal.pcbi.1006930 (PMC6457559; doi:10.1371/journal.pcbi.1006930)

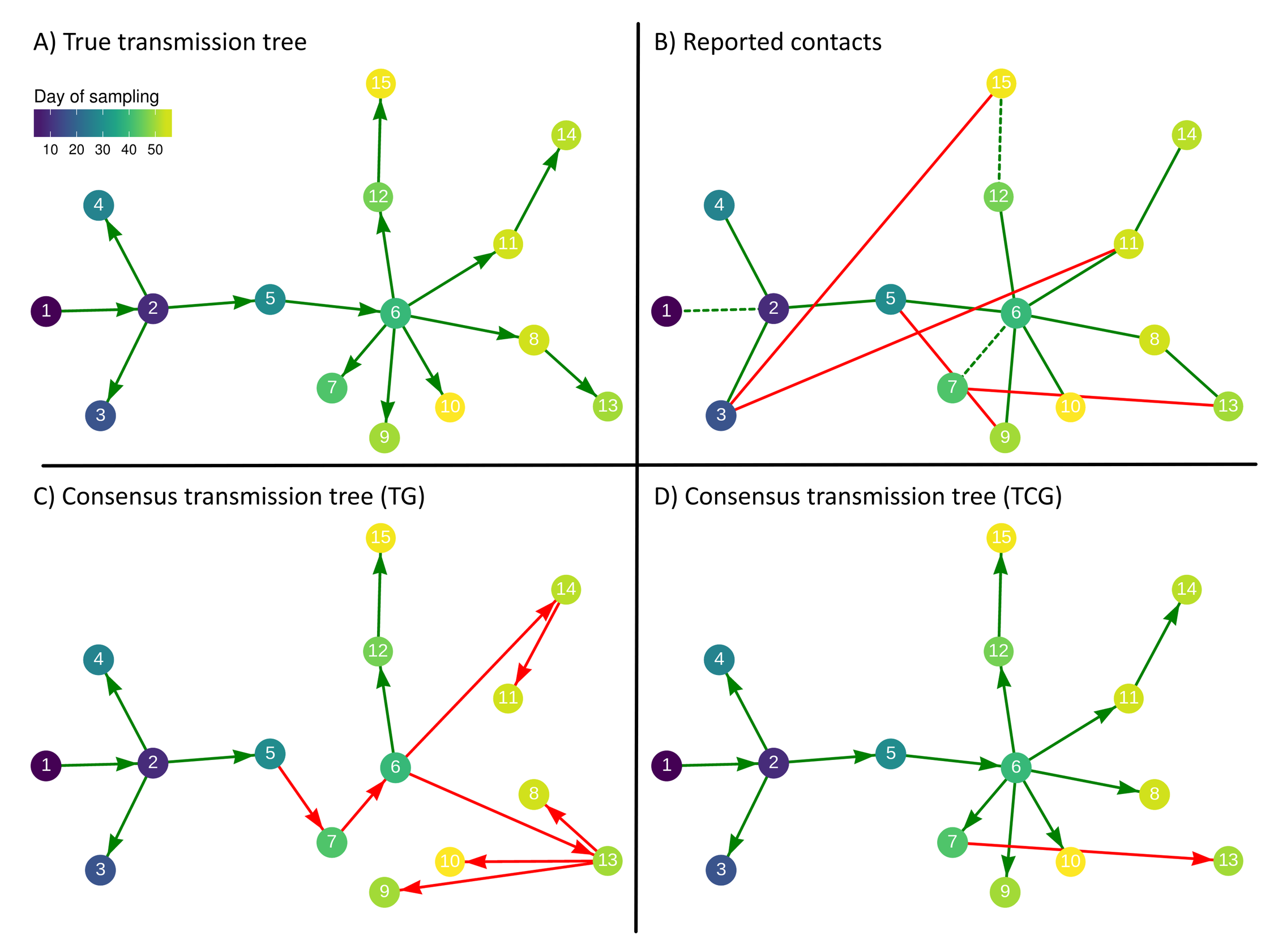

Supplement: S1 Fig — A) An Ebola-like outbreak of 15 cases was simulated in a susceptible population of 50 susceptible individuals. B) A contact network was simulated with a reporting coverage ε of 0.8 and a non-infectious contact probability λ of 0.1. Solid lines represent reported contacts; green lines correspond to transmission pairs, red lines to non-transmission pairs. Dashed green lines represent contacts between transmission pairs that were not reported. C) The outbreak was reconstructed using temporal and genomic data, and the consensus transmission tree, describing the modal posterior infector for each case, determined. Green lines correspond to correctly inferred ancestries, red lines to incorrectly inferred ancestries. The accuracy of outbreak reconstruction was 46%. D) The outbreak was reconstructed using temporal, genomic and contact data, with an accuracy of 94%. (TIF) [file pcbi.1006930.s004.tif]

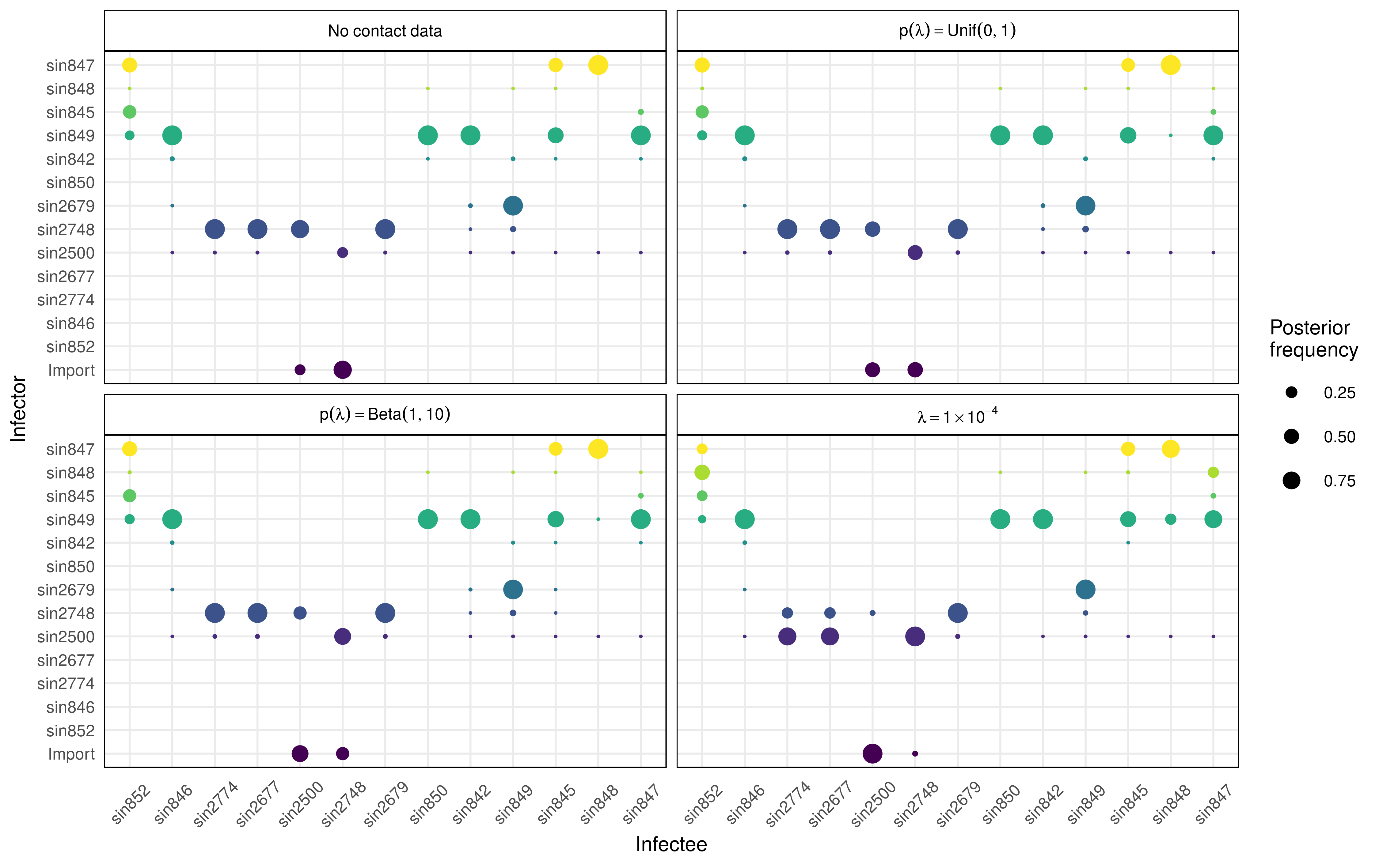

Supplement: S6 Fig — Columns represent sampled cases in the outbreak, rows represent potential sampled infectors. The size of each circle represents the posterior frequency of a given infector-infectee pair. (TIF) [file pcbi.1006930.s009.tif]
