# Supplementary material for: Bayesian inference of transmission chains using timing of symptoms, pathogen genomes and contact data
Source: PLoS Comput Biol. 2019 Mar 29;15(3):e1006930. doi: 10.1371/journal.pcbi.1006930 (PMC6457559; doi:10.1371/journal.pcbi.1006930)
Supplement: S3 Text — (DOCX) [file pcbi.1006930.s003.docx]

**S3 Text. Deriving the number of non-infectious contacts per person from the non-infectious contact probability.**

*λ* is defined as the probability of contact between two sampled individuals in an outbreak that do not constitute a transmission pair. *ψ* is defined as the expected number of non-infectious contacts per person. *ψ* can be derived from *λ* given knowledge of the number of sampled individuals N and the number of imported cases I.

The total number of possible contacts between N individuals is given by:

$$\frac{N(N-1)}{2}$$

Of these, N – I constitute a transmission pair, as every import reduces the number of transmission pairs by one. The total number of potential non-infectious contacts is therefore given by:

$$\frac{N(N-1)}{2}-(N-I)$$

$$\frac{N^{2}-3N+2I}{2}$$

As each contact involves two individuals, the average number of potential non-infectious contacts *per person* is then given by:

$$\frac{N^{2}-3N+2I}{2}\times\frac{2}{N}$$

$$N-3+\frac{2I}{N}$$

Finally, this term is multiplied by the probability of non-infectious contacts occurring, *λ*, to give the expected number of non-infectious contacts per person:

$$\Psi=\left( N-3+\frac{2I}{N} \right)\lambda$$
